# Congenital Fibrolipoma with Hamartomatous Changes of the Internal Jugular Vein: First Published Case Report

**DOI:** 10.14797/mdcvj.1519

**Published:** 2024-12-26

**Authors:** Samer Regal, Tamer A. Khafagy, Mohamed S. AbdelGawad, Ehab M. Saad, Ahmed A. Ali

**Affiliations:** 1 Cardiothoracic and Vascular Surgery Center, University Hospital, Mansoura University, Dakahliya, Egypt

**Keywords:** aneurysmal internal jugular vein, fibrolipoma, fibrofatty degeneration, hamartomatous wall morphology

## Abstract

A 25-year-old female presented with a congenital painless growing mass on the right side of her neck with symptoms of tinnitus and difficulty breathing. Imaging revealed an aneurysm of the internal jugular vein reaching a maximum diameter of 9.2 cm, shifting the trachea and right thyroid lobe to the left side. Simple excision was sufficient to treat compression symptoms and prevent potential thrombosis and embolism. This is the first reported case of aneurysmal internal jugular vein with fibrofatty degeneration and hamartomatous wall morphology associated with compression symptoms.

## Introduction

Fibrolipomas are benign soft tissue tumors comprising mature adipose tissue interlaced with fibrous tissue.^1,2^ They are relatively rare in vascular structures, and occurrences within major veins are particularly unusual. Among vascular anomalies, venous malformations involving fibrofatty degeneration are rarely reported, with most cases involving superficial or minor vessels rather than central veins such as the internal jugular vein (IJV).^3^

Hamartomatous changes in the IJV are exceedingly rare because hamartomas are typically congenital anomalies characterized by the disorganized growth of normal tissue in its usual location. Such vascular abnormalities can exert compressive symptoms due to their mass effect on surrounding structures, potentially causing functional disturbances or aesthetic concerns.^4^

This case report presents the first documented instance of a fibrolipoma with hamartomatous changes in the IJV. Here, we aim to contribute to the limited knowledge of vascular fibrolipomas with rare morphological presentations, underlining both the diagnostic approach and effective surgical management for alleviating symptoms and preventing complications.

The case report has been written following the CARE guidelines.^5^

## Case

A 25-year-old female presented with a congenital painless growing mass on the right side of her neck. She reported worsening tinnitus and episodic difficulty breathing but no pain or other symptoms. Physical examination revealed a prominent soft mass that was mobile and nontender upon palpation. There were no visible skin changes overlying the mass.

**Figure 1:**
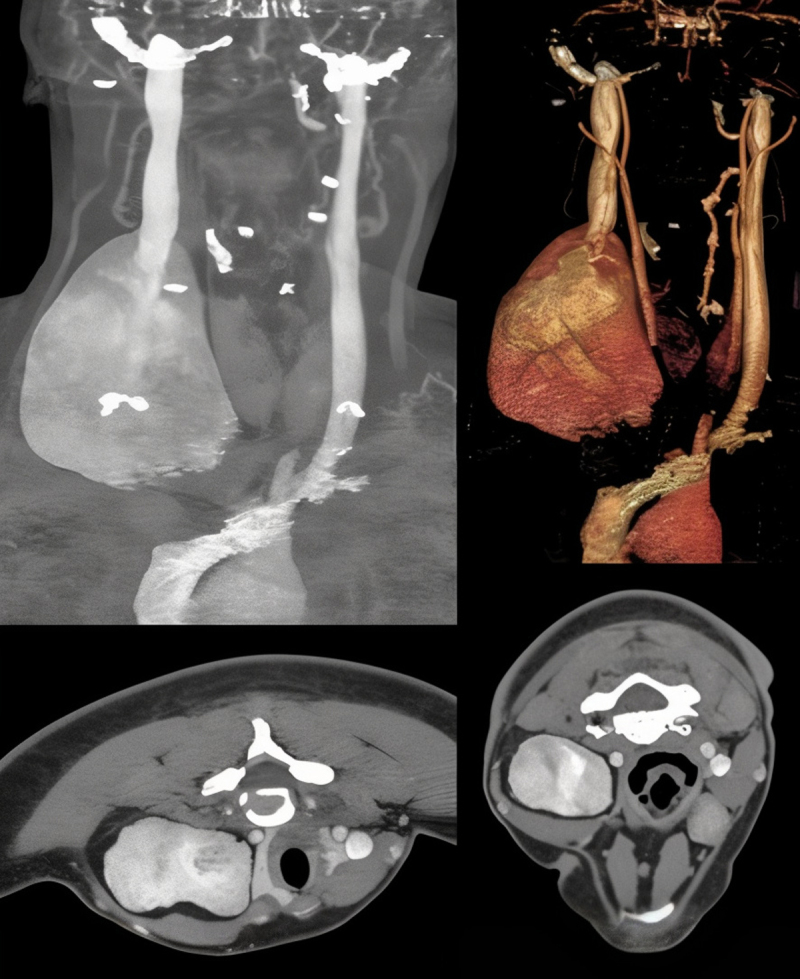
Preoperative computed tomography cerebral venography imaging of the internal jugular vein aneurysm.

Initial duplex ultrasound revealed an aneurysmal dilation of the IJV. Further imaging with contrast-enhanced computed tomography (CT) and magnetic resonance imaging (MRI) demonstrated an aneurysmal proximal IJV reaching a maximum diameter of 9.2 cm. This aneurysmal dilation caused a shift of the trachea and right thyroid lobe to the left side, with stretching of the strap muscles along the anterior surface. No intraluminal thrombus or other vascular abnormalities were detected on imaging (Figure 1).

The patient was counseled regarding potential treatment options, including simple excision, excision with vein reconstruction, aneurysmorrhaphy, and endovascular coiling. After a comprehensive discussion, a decision was made to proceed with simple excision of the aneurysmal segment with follow-up for additional procedures if needed. A consent was taken from the patient.

**Figure 2:**
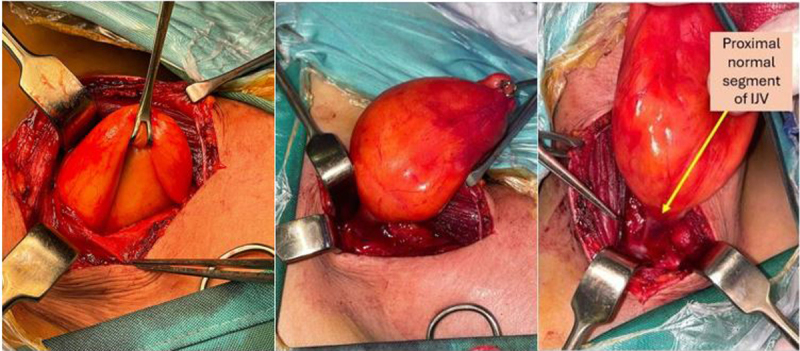
Intraoperative presentation of the internal jugular vein (IJV) aneurysm, and excising the vein up to the proximal segment of IJV. Arrow denotes the proximal normal segment of the IJV.

Under general anesthesia, the patient was placed in the supine position with the neck extended and turned to the left side. A right neck incision was made along the anterior border of the sternocleidomastoid muscle. Upon exposure, the aneurysmal segment of the IJV was identified within the carotid sheath (Figure 2). The common carotid artery and vagus nerve were mobilized and protected to allow safe dissection of the IJV. Notably, the vein had transformed into a sac-like structure rather than a typical aneurysmal dilation.

**Figure 3:**
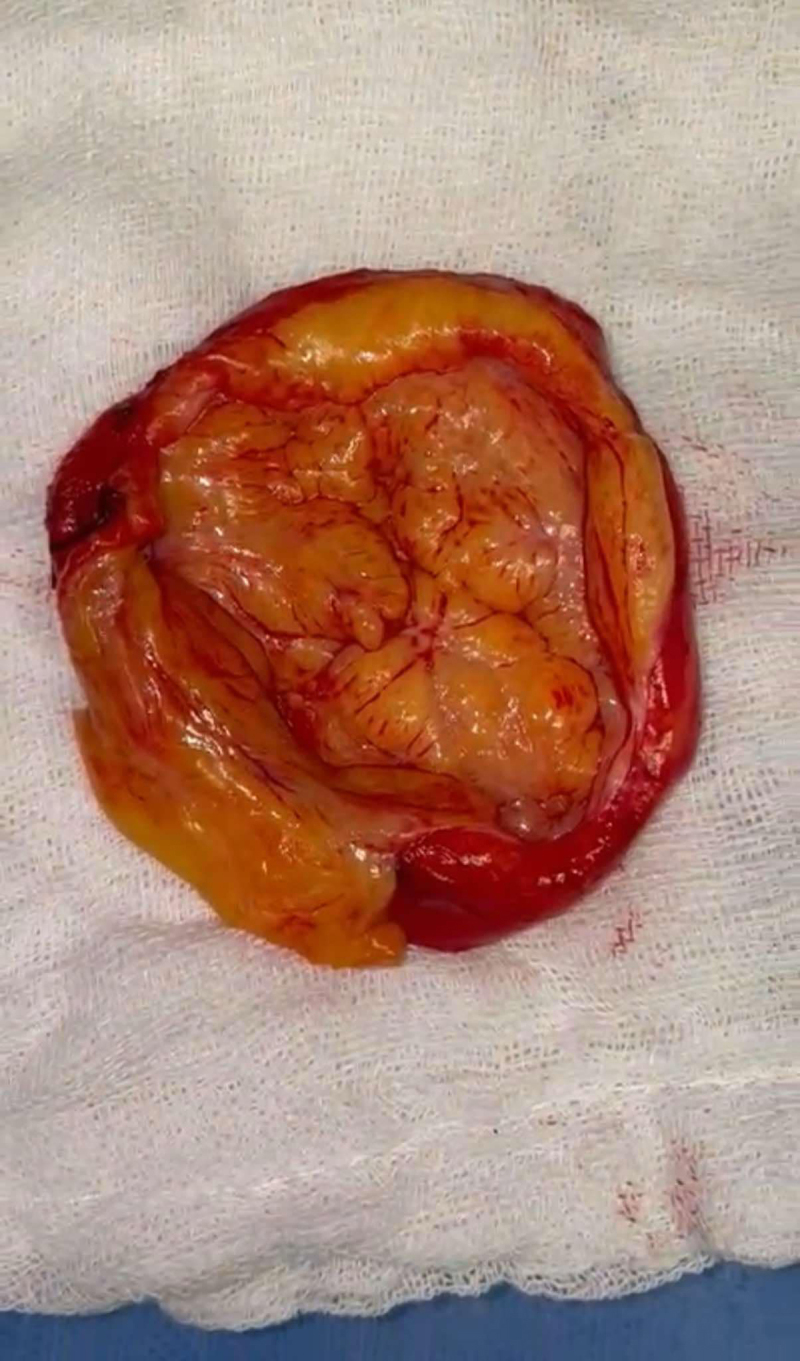
Content of the aneurysm.

The affected segment was excised without the need for reconstruction due to the presence of a normal ipsilateral external jugular vein and contralateral internal jugular vein (Figure 2). A representation of the transmitted respiratory pattern across the IJV and into the aneurysm can be seen in Video 1. Opening the aneurysm did not reveal any intraluminal affection (Figure 3, Video 2). Operative time was about 43 minutes with very minimal blood loss.

**Video 1:** Demonstration of transmitted respiratory flow within the aneurysmal segment of the internal jugular vein. The video highlights the movement of the venous wall synchronized with the patient’s breathing pattern; see also at https://youtube.com/shorts/gbH9sNRZDcM.

**Video 2:** Surgical opening of the aneurysmal segment. The video captures the intraoperative exploration of the aneurysm’s content, revealing the fibrolipomatous structure and confirming the absence of luminal occlusion; see also at https://youtube.com/shorts/tgAK2F-QTCY.

Histopathological examination of the resected specimen revealed tumor tissue formed of lobules of mature fat cells with intervening bundles of proliferating spindle cells as well as intervening hamartomatous fibromuscular-lined vascular spaces, which were not filled with blood but with thick collagen bundle formation. This resulted in a diagnosis of fibrolipoma with ectatic empty hamartomatous vascular space. There was no evidence of malignancy or intraluminal involvement.

The patient had an uneventful postoperative course and was discharged on the second day post-surgery. On 6-month follow-up, the patient reported complete resolution of tinnitus and improvement in breathing, with no residual compression symptoms.

## Discussion

Vascular malformations of the IJV are exceedingly rare, with most documented cases involving peripheral or superficial veins rather than central venous structures. Fibrolipomas, characterized by mature adipocytes interlaced with fibrous tissue, typically appear in subcutaneous or neural locations and are infrequent within major vascular structures such as the IJV.^1,2,3,6^ This case is unique due to the presence of fibrolipomatous growth with hamartomatous changes in the IJV with no luminal affection, a combination not previously documented.

While fibrolipomas in veins are rare, similar cases of benign intravascular tumors have been reported. For instance, an intravascular lipoma in the IJV was described in a patient presenting with neck pain and arm swelling, confirmed by CT and MRI, with successful surgical excision confirming its benign nature.^7^ Another rare case involved a solitary fibrous tumor within the IJV that presented as a neck mass and necessitated surgical removal.^8^ In the femoral vein, an intravascular fibrolipoma was successfully resected, illustrating that fibrolipomas, when symptomatic, benefit from surgical intervention.^6^ These examples highlight the diversity of benign intravascular lesions, which are essential to consider when diagnosing atypical neck masses. In one case where the fibrolipoma did not involve the main vessels, the authors showed the potential for fibrolipomas to grow to significant sizes and cause compressive symptoms, emphasizing the importance of early diagnosis and management.^9^

Numerous studies have reported the presence of lipomas in central veins, ie, the superior vena cava and brachiocephalic vein, and some have reported the occurrence of such lesions in the inferior vena cava and the renal vein.^10,11,12^ However, they were all lipomas or hemangiomas with luminal affection.

The differential diagnosis for painless compressive neck masses includes benign lipomas, hemangiomas, and, more rarely, venous aneurysms with fibrofatty degeneration. High-resolution imaging such as duplex ultrasound, CT, and MRI plays a crucial role in differentiating these entities. In our case, imaging revealed the mass’s size, vascular origin, and anatomical relationship to adjacent structures, which is essential for preoperative planning. Histopathology confirmed the diagnosis demonstrating fibrolipomatous tissue with hamartomatous features, underscoring the value of tissue examination for rare venous lesions.

In managing IJV fibrolipomas, simple excision is often sufficient to relieve compressive symptoms without necessitating reconstruction, especially when collateral venous drainage is intact. This approach effectively alleviates symptoms while minimizing surgical complexity and risks, as demonstrated in our patient. The benign nature of fibrolipomas allows for straightforward surgical resection with a favorable prognosis and low recurrence rates, as similarly reported in other cases of intravascular lipomas and fibrolipomas.^1,6,7,8,9,10,11,12,13^

Most literature regarding vascular malformations with fibrofatty changes in major veins is limited to sporadic reports involving smaller veins or superficial structures. A few cases have documented fibrolipomatous changes within soft tissues surrounding major vascular structures, yet none have involved the IJV or presented with the distinct histopathological combination observed here.

## Conclusion

This case report documents the first known instance of a fibrolipoma with hamartomatous changes in the internal jugular vein presenting as aneurysmal dilation with compressive symptoms. Simple excision proved effective in resolving symptoms and preventing complications without requiring reconstruction. The case contributes novel insights into the presentation, management, and outcomes of rare venous fibrolipomas and highlights the value of histopathological examination for confirming diagnosis. Future similar cases may benefit from a similar surgical approach, tailored to each patient’s unique anatomical and symptomatic profile.
